# On *Afromantispa* and *Mantispa* (Insecta, Neuroptera, Mantispidae): elucidating generic boundaries

**DOI:** 10.3897/zookeys.523.6068

**Published:** 2015-09-28

**Authors:** Louwtjie P. Snyman, Catherine L. Sole, Michael Ohl

**Affiliations:** 1 Department of Zoology and Entomology, University of Pretoria, Pretoria 0002, South Africa; 2 Museum für Naturkunde Berlin, Invalidenstr. 43, 10115 Berlin, Germany

**Keywords:** Mantispidae, *Afromantispa*, *Mantispa*, Afrotropics, Palearctic

## Abstract

The genus *Afromantispa* Snyman & Ohl, 2012 was recently synonymised with *Mantispa* Illiger, 1798 by Monserrat (2014). Here morphological evidence is presented in support of restoring the genus *Afromantispa* **stat. rev.** to its previous status as a valid and morphologically distinct genus. Twelve new combinations (**comb. n.**) are proposed as species of *Afromantispa* including three new synonyms.

## Introduction

Mantispidae (Leach, 1815) is a small cosmopolitan family in the very diverse order Neuroptera. The former is characterised by an elongated prothorax, elongated procoxa protruding from the anterior pronotal margin and conspicuous raptorial forelegs. Recently, one of the genera, *Mantispa* Illiger, 1798 has been the focus of taxonomic studies (Snyman et al. 2012; Monserrat 2014). *Mantispa* was originally described by Illiger (1978) and quickly became the most speciose genus with a cosmopolitan distribution. Studies by Lambkin (1986a; b), Hoffman (2002), Machado and Rafael (2010) and Snyman et al. (2012) excluded *Mantispa*’s distribution from much of the world and consequently *Mantispa* is no longer thought to occur in the Neotropics, Nearctic, Afrotropics or Australasia. *Mantispa*, according to the morphology of the type species *Mantispa pagana* (Fabricius, 1775) (synonymised with *Mantispa styriaca* (Poda, 1761)), is thus probably a small genus from the Palearctic. As the previously mentioned studies focused on the fauna elsewhere the generic boundaries between *Mantispa* and other similar groups have remained poorly understood.

In their study on the Afrotropical mantispid genera, Snyman et al. (2012) proposed that the majority of the *Mantispa*-like species from the Afrotropics and south western parts of Europe can be defined as a separate genus and consequently described *Afromantispa*. The authors unfortunately did not provide a list of species belonging to the newly erected genus claiming it would be best left until a full revision of the genus could be launched. It appears that this might have caused some additional confusion.

Monserrat (2014) synonymised *Afromantispa* with *Mantispa* in a study only focussing on the local fauna of the Iberian Peninsula and Balearic Islands. A new species *Mantispa incorrupta* was also described. Additionally, the author synonymised *Sagittalata* Handschin, 1959 with *Mantispa*. The status of *Sagittalata* is currently still in dispute and not well understood.

*Afromantispa*, *Mantispa* and *Sagittalata* are quite difficult to distinguish, but several morphological characters do support separation of the genera. Adding to the difficulty is that there is a distribution overlap between species from both genera in southern and western Europe. The antennae, prothorax, mesothorax, pterostigma, and fifth tergite are morphologically different between members of the genera (Table 1). *Mantispa* are represented by only two species that can confidently be placed in the genus (supplementary files: Appendix II). The status of *Afromantispa* (Snyman & Ohl, 2012) is hereby restored as a genus morphologically distinct from *Mantispa*, and a list of the species that belong to *Afromantispa* is provided. This study thus aims to elucidate the boundaries between these two genera.

**Table 1.** Morphological characters separating *Mantispa* and *Afromantispa*.

	*Afromantispa*	*Mantispa*
Short stout setae on occiput (Fig. 1f. (i))		●
Pale band in distal third of the antennae (Fig. 1b. (i))	●	
Granulated prothorax (Fig. 1a–d; 2e–f)	●	
Short stout setae on mesothorax (Fig. 1f. (ii))		●
Bicoloured pterostigma (Fig. 2a. (i); b. (i))	●	
Enlarged fifth male tergite (Fig. 3c. (i))	●	

## Material and methods

The specimens used in this study are housed at the following institutions:

AMG Albany Museum, Grahamstown, South Africa

BMNH The Natural History Museum, London, Great Britain

HUAC Personal collection H. and U Aspöck, Vienna, Austria

MNHN Museum National d’Histoire Naturelle, Paris, France

MRAC Musee Royal de l’Afrique Centrale, Tervuren, Belgium

MZBS Museo Zoologia, Barcelona, Spain

NHMB Naturhistorisches Museum, Basel, Switzerland

OUM University Museum, Oxford, Great Britain

SANC South Arfican National Collection, Roodeplaat, South Africa

VMC Personal collection V. Monserrat, Madrid, Spain

ZMB Museum für Naturkunde, Berlin, Germany

Photos were taken with either a Canon 500D equipped with a 100mm Canon macro lens or with a Leica Z16 APOA camera setup.

All type specimens that are not housed at MRAC and ZMB were studied using high resolution photographs provided by ZMB (supplementary files: Appendix IV). Adult morphological terminology follows that of Lambkin (1986a; b).

## Taxonomic amendments

### Morphological overview

**Figure 1.**
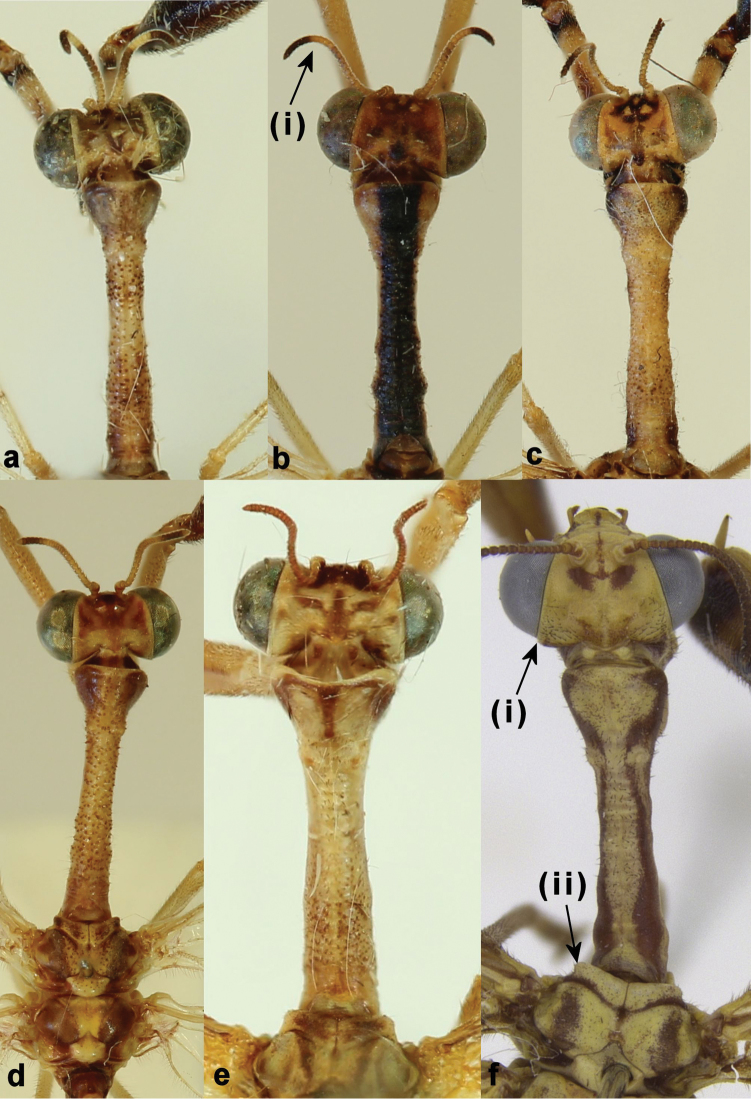
Prothorax in dorsal view of **a** *Afromantispa capeneri* (Handschin, 1959) **b** *Afromantispa moucheti* (Navás, 1925) **c** *Afromantispa nana* (Erichson, 1839) **d** *Afromantispa tenella* (Erichson, 1839) **e** *Mantispa styriaca* **f** *Mantispa aphavexelte* (photo credits: **a–d** Johan Saayman).

**Figure 2.**
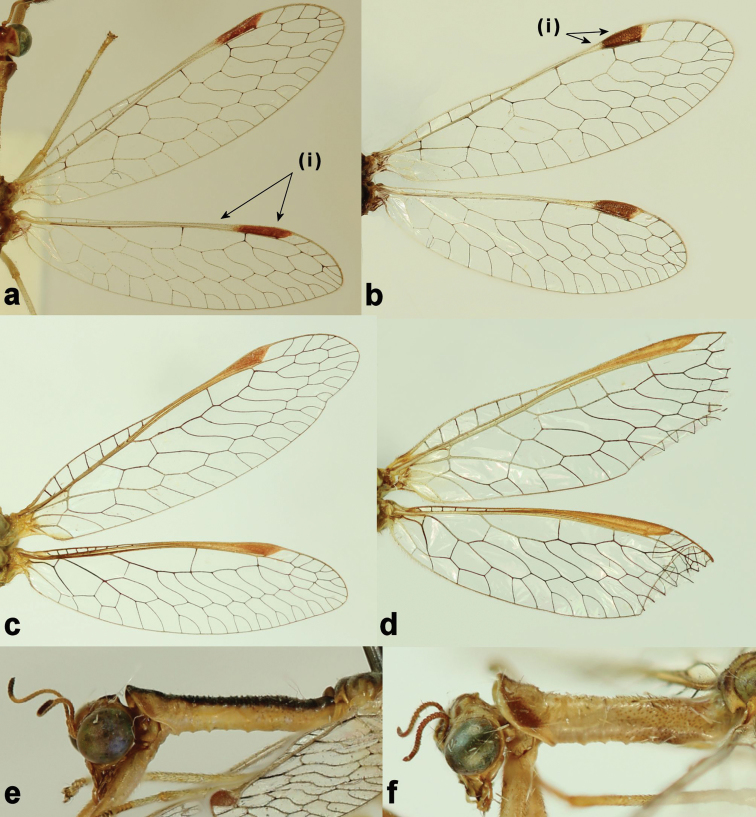
Right wings of **a** *Afromantispa tenella* **b** *Afromantispa moucheti* **c** *Mantispa styriaca* **d** *Mantispa aphavexelte*. Prothorax in lateral view of **e** *Afromantispa moucheti* **f** *Mantispa styriaca* (photo credits **a–b; e–f** Johan Saayman).

**Figure 3.**
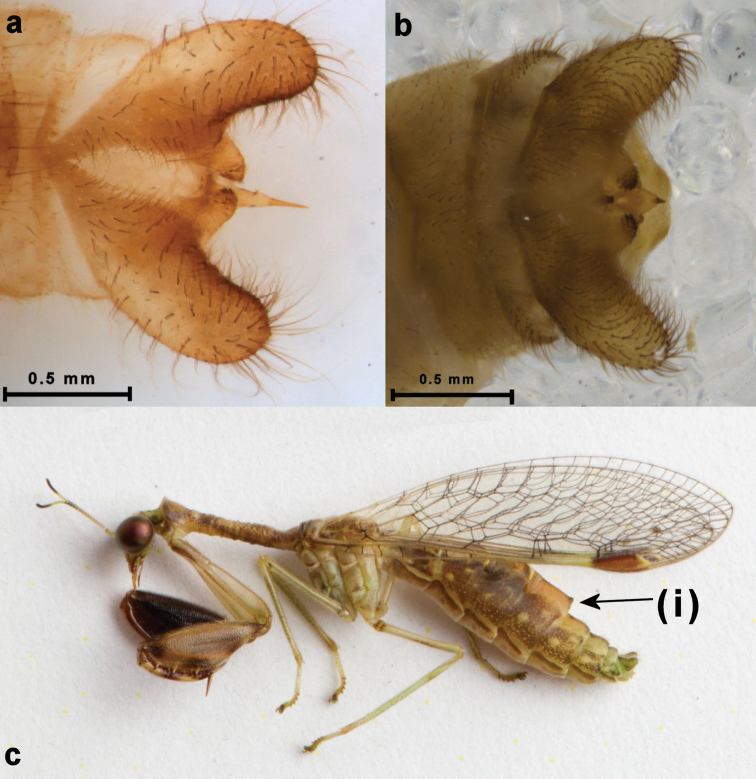
Terminalia of **a** *Afromantispa zonaria* (Navás, 1925) – type specimen from MRAC **b** *Mantispa aphavexelte*. Freshly killed *Afromantispa tenella* indicating enlarged fifth male tergite (photo credits: **a** Ludwig Eksteen, **c** Morgan Trimble).

*Head*: The flagella of the genera are quite similar in appearance but all species of *Afromantispa* have a pale band in the distal third of the flagella, this character is not shared by the species of *Mantispa*. The band is even distinct in *Afromantispa* species with light yellowish flagella. In the latter, the band is then presented by a few dark antennules prior to the band so it remains visible (Fig. 1b; d) (Monserrat 2014: fig. 43). In *Mantispa*, the occiput is covered by short stout setae dorsolaterally (Fig. 1f); this feature is not present in the *Afromantispa* species studied. The rest of the head capsule is very similar between the taxa. The prothorax dorsum of *Afromantispa* is always covered in granules and setae, where the *Mantispa* species lack granules, even if small pigmentation “dots” are visible at the origin of the setae on *Mantispa styriaca* (Fig. 1a–f) (Monserrat 2014: fig. 23–24). Peculiarly, a region in the lateral mid-zone of the prothorax of *Afromantispa* always lacks granules (Fig. 2e; f) (Monserrat 2014: fig: 45). *Mantispa* in turn have short stout setae on the dorsum of the mesothorax, which is lacking in *Afromantispa* (Fig. 1f). The wing venation of both genera is very similar in structure except for features pertaining to the pterostigma (Fig. 2a–d). The costal space in *Mantispa* seems slightly larger than in *Afromantispa*, but it can vary. The pterostigma in *Mantispa* however, is different. The subcosta and radius of *Afromantispa* is always pale/yellowish in colouration up to or just distal to midway of radial cell 2. Thereafter, the pterostigma commences. The proximal end of the pterostigma is the same pale colouration of the subcosta and radius veins. The centre of the large distal half is always reddish or dark in colouration flanked by a thin yellowish margin until meeting the veins. The pterostigma of some species might be slightly truncated and anteriorly rounded (Fig. 2b). *Mantispa* in turn always have a reddish monocoloured pterostigma (Fig. 2c–d). The terminalia of both genera are similar (Fig. 3a–b) in structure where variation on ectoproct size and length is common in *Afromantispa*. Both genera have an extrusible gland present between tergite V and VI (Eltringham 1932). Tergite V of *Afromantispa* is conspicuously enlarged, especially in fresh specimens (Fig. 3c). From various photos of live *Mantispa styriaca* and *Mantispa aphavexelte* Aspöck & Aspöck, 1994, including those of Monserrat (2014), it was determined that this tergite is not as prominent in *Mantispa*.

The elongated line on the procoxa of *Sagittalata* suggested by Snyman et al. (2012) was considered as a weak character by the authors. *Sagittalata* lacks the greatly enlarged gland present between the V and VI tergites. In addition, species from *Sagittalata* have a dorsally enlarged inner flange on the caudal apex of the gonocoxites as illustrated by Poivre (1981a (fig. 2 J; 4E); 1981b (fig. 1T; 3X; 6R; 7S); 1983 (fig. 3 H, L)). The cusp on the anterior margin of the pronotum lacks short stout setae which are present in *Mantispa*. The pronotal dorsum lacks short stout setae that are present in *Mantispa*, but may have a few sparsely distributed setae. The mesothorax completely lacks prominent setae and is either glaborous or pubescent (velvet appearance) which is also different in *Mantispa*. *Sagittalata* might be forming part of the previously synonymised genus *Mantispilla*, thus, changing the taxonomic status of the genus in this paper only to be considered moot in subsequent publications seems illogical (Snyman et al. in prep). The current synonymy suggested by Monserrat (2014) are considered valid but are excluded from the species list in the Suppl. material 1.

## Discussion

These two genera are possibly quite closely related and therefore present several confusing morphological characteristics. The suggestion by Monserrat (2014) to synonymise the genera has cascading effects on the taxonomy of Mantispinae. Even though western Europe is not specifically rich in mantispid species, these genera are not confined to that area. The suggested synonymy by Monserrat (2014) means that *Mantispa* will again include 144 species spanning Africa, Europe, Asia and some Australasian islands (numbers from Ohl 2004). This might be possible, but should be approached with caution and include a substantially larger number of species than what was included by the author. Monserrat (2014) further only considered species formally recorded from Spain, where a much larger scope should have been included.

The following species all conform to the characters proposed in this study and are consequently regarded as belonging to *Afromantispa*: *capeneri* (Handschin), comb. n., *dispersa* (Navás), comb. n., *incorrupta* (Monserrat), comb. n., *meadewaldina* (Navás), comb. n., *moucheti* (Navás), comb. n., *nana* (Erichson), comb. n., *nanyukina* (Navás), comb. n. *natalensis* (Navás), comb. n., *navasi* (Handschin), comb. n., *verruculata* (Navás), comb. n., *zonaria* (Navás), comb. n., *zonata* (Navás), comb. n. *Afromantispa arabica* (Navás, 1914f), syn. n. is a new synonym of *Afromantispa nana* (Erichson, 1839). *Afromantispa variolosa* (Navás, 1914d), syn. n. is a new synonym of *Afromantispa tenella*. *Afromantispa schoutedeni* (Navás, 1929), syn. n. is a new synonym of *Afromantispa moucheti* (Navás) (supplementary files: Suppl. material 1 I). Several other species have been described with a distribution in the Afrotropics (supplementary files: Suppl. material 1 III). The type specimens of these species have not yet been studied and the placement of the species remains uncertain. The distribution of the genus suggests that these might belong to *Afromantispa* or another less likely, another Afrotropical genus. Their current placement in *Mantispa* is most likely a historical one and possibly erroneous. Until the type specimens are studied, the names should remain in *Mantispa*.

This study confidently presents enough data for the separation of *Afromantispa* and *Mantispa*. Current integrative studies including the authors of this study are ongoing focussing on the elucidation of the world genera of the mantispines.

## Supplementary materials

Supplementary material 1 List and table of species names, updated from Ohl (2004) Data type: species data This dataset is made available under the Open Database License (http://opendatacommons.org/licenses/odbl/1.0/). The Open Database License (ODbL) is a license agreement intended to allow users to freely share, modify, and use this Dataset while maintaining this same freedom for others, provided that the original source and author(s) are credited.Louwtjie P. Snyman, Catherine L. Sole, Michael Ohl
