# Supplementary material for: On Afromantispa and Mantispa (Insecta, Neuroptera, Mantispidae): elucidating generic boundaries
Source: Zookeys. 2015 Sep 28;(523):89–97. doi: 10.3897/zookeys.523.6068 (PMC4602298; doi:10.3897/zookeys.523.6068)
Supplement: Supplementary material 1 — List and table of species names, updated from Ohl (2004) [file zookeys-523-089-s001.docx]

**Supplementary Material**

Names updated from Ohl 2004. Museum abbreviations taken from Ohl (2004). Referencing facilitated by reference manager Mendeley Desktop 1.13.8 (<https://www.mendeley.com> _Accessed 14/05/2015)

**Appendix I:** Validated species considered as belonging to *Afromantispa*

**Appendix II:** Validated species considered as belonging to *Mantispa*

**Appendix III:** Unplaced/Unstudied *Mantispa* species recorded from the Afrotropics and therefore the current classification is considered doubtful

**Appendix IV:** List of specimens used in this study

References of Appendices

**Appendix I**

All of the following species share the above mentioned characters of *Afromantispa* and is considered to be part of *Afromantispa.* Photographs of the type specimens were studied and subsequently transferred to *Afromantispa*.

***Afromantispa*** (Snyman & Ohl 2012)

*Afromantispa* Snyman & Ohl in Snyman et al. 2012. Type species: Afromantispa tenella Erichson, 1839. Syntypes: sex unknown, South Africa (ZMB).

*Afromantispa capeneri* Handschin **comb. nov.**

*Mantispa capeneri* Handschin, 1959. Syntypes: male, South Africa (MRAC).

Distribution: Afrotropical: South Africa.

*A. dispersa* (Navás) **comb. nov.**

*Mantispilla dispersa* Navás. 1914e. Syntypes: sex unknown, Malawi, South Africa, Zimbabwe (BMNH). Originally as *Mantispa tenella var, dispersa* Erichson according to Handschin 1959.

Distribution: Afrotropical: Malawi, South Africa, Zimbabwe

*A. incorrupta* Monserrat **comb. nov.**

*Mantispa incorrupta* Monserrat, 2014. Holotype: Holotype: male, Toledo, Spain (VM). Paratypes: two males, three females (VM), one male (MNCNM), Toledo, Spain.

Distribution: Palearctic: Spain

*A. meadewaldina* (Navás) **comb. nov.**

*Mantispilla meadewaldina* Navás, 1914e. Holotype (or syntypes): sex unknown, Nigeria (BMNH).

Distribution: Afrotropical: Nigeria

*A. moucheti* (Navás) **comb. nov.**

*Mantispilla moucheti* Navás, 1925 [as *Moucheti* [sic], incorrect original capitalization]. Holotype (or syntypes): female, DR Congo (MRAC).

*Mantispilla schoutedeni* Navás 1929 [as *Schoutedeni* [sic], incorrect original capitalization]. Holotype (or syntypes): sex not indicated, DR Congo (MRAC) **syn. nov.**

Distribution: Afrotropical: DR Congo. Afrotropical: DR Congo, South Africa, Tanzania.

*A. nana* Erichson **comb. nov.**

*Mantispa nana* Erichson, 1839. Syntypes: female, Sudan (ZMB MCZ).

*Necyla bonhourei* Navás, 1922 [as *Bonhourei* [sic], incorrect original capitalization]. Holotype (or syntypes): sex unknown, Djibouti (MNHN). Synonymised with *M. nana* by Handschin 1959.

*Necyla arabica* Navás, 1914b. Holotype (or syntypes): sex unknown, Saudi Arabia (BMNH) **syn. nov.**

Distribution: Afrotropical: Eritrea, Congo, Djibouti, South Africa, Sudan, Upper Volta. Palaearctic: Arabian Peninsula.

*A. nanyukina* Navás **comb. nov.**

*Mantispilla nanyukina* Navás, 1933b. Holotype (or syntypes): female, Kenya

(MNHN according to original description, MZBS according to Monserrat, 1985.

Distribution: Afrotropical: Kenya.

*A. natalensis* Navás **comb. nov.**

*Necyla natalensis* Navás, 1914a. Holotype (or syntypes): male, South Africa (OUM).

Distribution: Afrotropical: South Africa.

*A. navasi* Handschin **comb. nov.**

*Mantispilla umbripennis* Navás, 1914g. Holotype (or syntypes): female, DR Congo (MRAC). As *Mantispa umbripennis* a junior secondary homonym of *Mantispa umbripennis* Walker, 1860.

*Mantispa navasi* Handschin, 1960. Replacement name for *Mantispa umbripennis* (Navás 1914f), a junior secondary homonym of *Mantispa umbripennis* Walker, 1860.

Distribution: Afrotropical: South Africa, DR Congo, Tanzania.

*A. tenella* Erichson

*Mantispa tenella* Erichson, 1839. Syntypes: sex unknown, South Africa (ZMB).

*Mantispa variolosa* Navás, 1914d. Holotype (or syntypes): sex unknown "Oceanía"(BMNH) **syn. nov.**

*Mantispilla tenera* Navás, 1914h. Holotype (or syntypes): sex unknown, Kenya (MNHN). Synonymised with *Mantispa tenella* by Handschin, 1959.

*Mantispa axillata* Navás, 1936a: sex not indicated. Holotype (or syntypes): sex unknown, DR Congo (MRAC). Synonymised with *Mantispa tenella* by Handschin, 1959.

Distribution: Afrotropical: DR Congo, Ivory Coast, Kenya, Malawi, Nigeria, "Italian Somaliland " (Somalia, Djibouti, or Ethiopia), South Africa, Tanzania, Zimbabwe.

*A. verruculata* (Navás) **comb. nov.**

*Mantispilla verruculata* Navás, 1914h. Holotype (or syntypes): sex unknown, "British East Africa" (MNHN).

*Mantispilla maynei* Navás, 1929a [as *Maynei* [sic], incorrect original capitalization]. Syntypes: sex unknown, DR Congo (MRAC). Synonymised by Handschin 1959

Distribution: Afrotropical: DR Congo, Kenya, South Africa, Uganda.

*A. zonaria* (Navás) **comb. nov.**

*Mantispa zonaria* Navás, 1925. Holotype: male, DR Congo (BMNH MRAC).

Distribution: Afrotropical: Cameroon, DR Kongo, Ivory Coast, Tanzania

*A. zonata* (Navás) **comb. nov.**

*Mantispa zonata* Navás, 1923. Holotype (or syntypes): sex unknown, Gabon (MNHN).

Distribution: Afrotropical: Gaboon

**Appendix II**

Validated species considered as belonging to *Mantispa*

***Mantispa*** Illiger

*Mantispa* Illiger in Kugelann, 1798. Type species: *Mantis pagana* Fabricius, 1775 (= Raphidia styriaca (Poda 1761), by monotypy.

Amycla Rafinesque, 1815. Unjustified emendation of *Mantispa* Illiger in Kugelann, 1798. Amycla was considered an emendation of *Mantispa* Illiger in Kugelann, 1798, by Neave, 1939.

*Mantispilla* Enderlein, 1910 (as subgenus of *Mantispa* Illiger in Kugelann, 1798). Type species: *Mantispa indica* Westwood, 1852, by original designation. Synonymized with *Mantispa* by Penny, 1982b.

*Sagittalata* Handschin, 1959. Type species: *Mantispilla hilaris* Navás, 1925 (as "*Sagittalata hilaris* (Navás 1924 [sic])"), by original designation. Synonymised with *Mantispa* by Monserrat 2014.

*Perlamantispa* (Handschin, 1960). Type species: *Mantis perla* Pallas, 1772 (as "*Mantispa perla*"), by original designation. Synonymised with *Sagittalata* by Snyman et al., 2012.

Distribution: Too many doubtful records, known distribution in the Paleacrtic.

The following species share the above mentioned characters of *Mantispa* and lack the characters shared by *Afromantispa* and are validated as *Mantispa.*

*M. aphavexelte* Aspöck & Aspöck

*Mantispa aphavexelte* Aspöck and Aspöck, 1994. Holotype: male, Greece (HUAC).

Distribution: Palaearctic: southern Europe, Turkey, Caucasus area, Kazakhstan, Mongolia, China?

*M. styriaca* (Poda)

*Raphidia styriaca* Poda, 1761 [as *Styriaca* [sic], incorrect original capitalization]. Holotype (or syntypes): sex unknown, Austria (depository unknown).

*Raphidia mantispa* Scopoli, 1763 [as *Raphidia Mantispa* [sic]; incorrect original capitalization]. Holotype (or syntypes): sex unknown, "Carniolia". Synonymised with *Mantis pagana* Fabricius (now in *Mantispa*) by Oliver, 1797.

*Mantis pagana* Fabricius, 1775. Holotype (or syntypes): sex unknown, France (depository unknown). Synonymised with *Mantispa styriaca* by Aspöck et al., 1980.

*Mantispa hauseri* Poivre, 1982. Holotype: female, Croatia (MHNG). Synonymised by Aspöck and Aspöck, 1994.

*Mantispa kononenkoi* Makarkin, 1985. Holotype: male, Russia (ZIL). Synonymised with *Mantispa styriaca* by Makarkin, 1990.

Distribution: Palaearctic: widespread in Eurasia south of 50°N, Morocco.

**Appendix III**

Unplaced/Unstudied *Mantispa* species recorded from the Afrotropics and therefore the current classification is considered doubtful and in need of revision

*M. basilei* (Navás)

*Mantispilla basilei* Navás, 1930b [as *Basilei* [sic], incorrect original capitalization]. Holotype: female, Ethiopia (MCSN).

Distribution: Afrotropical: Ethiopia.

*M. castaneipennis* Esben-Petersen

*Mantispa* (*Mantispilla*) *castaneipennis* Esben-Petersen, 1917. Holotype: female, South Africa (ZMUH).

Distribution: Afrotropical: South Africa.

*M. centenaria* Esben-Petersen

*Mantispa* (*Mantispilla*) *centenaria* Esben-Petersen, 1917. Holotype: male, South Africa (ZMUC).

Distribution: Afrotropical: South Africa.

*M. delicata* (Navás)

*Mantispilla delicata* Navás, 1914e. Holotype (or syntypes): sex unknown, South Africa (BMNH).

Distribution: Afrotropical: South Africa.

*M. ellenbergeri* (Navás)

*Mantispilla ellenbergeri* Navás, 1927 female [as *Ellenbergeri* [sic], incorrect original capitalization]. Holotype (or syntypes): female, South Africa (MNHN).

Distribution: Afrotropical: South Africa.

*M. elpidica* (Navás)

*Mantispilla elpidica* Navás, 1914c. Holotype (or syntypes): sex unknown, South Africa (BMNH).

Distribution: Afrotropical: South Africa.

*M. fuscipennis* Erichson

*Mantispa fuscipennis* Erichson, 1839. Holotype: male, South Africa (ZMB).

Distribution: Afrotropical: South Africa, Tanzania.

*M. haematina* (Navás)

*Mantispilla haematina* Navás, 1914a. Holotype: female, Zimbabwe (OUM).

Distribution: Afrotropical: Zimbabwe.

*M. lutea* (Stitz)

*Mantispilla lutea* Stitz, 1913. Holotype: female, Ethiopia (ZMB).

Distribution: Afrotropical: Ethiopia.

*M. marshalli* (Navás)

*Mantispilla marshalli* Navás, 1914e [as *Marshalli* [sic], incorrect original

capitalization]. Holotype (or syntypes): sex unknown, Zimbabwe (BMNH).

Distribution: Afrotropical: Zimbabwe.

*M. nubila* (Stitz)

*Mantispilla nubila* Stitz, 1913. Holotype: female, Cameroon (ZMB).

Distribution: Afrotropical: Cameroon.

*M. phaeonota* Navás

*Mantispa phaeonota* Navás, 1933b. Holotype (or syntype): male, Kenya [erroneous locality - Madagascar considered as correct locality] (MNHN).

Distribution: Afrotropical: Madagascar (see annotation above).

*M. tessmanni* (Stitz)

*Mantispilla tessmanni* Stitz, 1913. Holotype: male, Equatorial Guinea (ZMB).

Distribution: Afrotropical: DR Congo, Equatorial Guinea.

*M. umbripennis* Walker

*Mantispa umbripennis* Walker, 1860. Holotype (or syntypes): sex unknown, South Africa (BMNH).

Distribution: Afrotropical: South Africa, Tanzania

**Appendix IV:**

| Original epithet | Current status | Type | Locality | Studied material | Depositry |
| --- | --- | --- | --- | --- | --- |
| *Afromantispa* |  |  |  |  |  |
| *arabica* | *A. nana* (syn.) | holotype, female | Saudi Arabia | photo | BMHN |
| *axillata* | *A. tenella* (syn.) | holotype, sex unknown | DR Congo | photo, pinned specimen | MRAC |
| *bonhourei* | *nana* (syn.) | type, sex unknown | Djibouti | photo | MNHN |
| *capeneri* | *A. capeneri* | syntype, female | Natal, South Africa | photo | NHMB |
| *capeneri* | *A. capeneri* | syntype, male | Natal, South Africa | photo | NHMB |
| *dispersa* | subsp. of *A. tenella* | syntype, male | Mashonaland, Zimbabwe | photo | BMNH? |
| *dispersa* | subsp. of *A. tenella* | syntype, female? | Oshogbo, Nigeria | photo | BMNH? |
| *incorrupta* | A. incorrupta | holotype | Spain | literature, (Monserrat 2014) | VMC |
| *maynei* | *A. verruculata* (syn.) | holotype, sex unknown | DR Congo | photo, pinned specimen | MRAC |
| *meadewaldina* | *A. meadewaldina* | holotype, female | Lagos, Nigeria | photo | BMHN |
| *moucheti* | *schoetedeni* **(syn. nov.)** | holotype, female | DR Congo | photo, pinned specimen | MRAC |
| *nana* | *A. nana* | syntype, male | Dongola, Sudan | photo, pinned specimen | ZMB |
| *nana* | *A. nana* | syntype, male? | Dongola, Sudan | photo, pinned specimen | ZMB |
| *nanyukina* | *A. nanyukina* | holotype, female | Nanyuki, Kenya | photo | MZBS |
| *natalensis* | *A. natalensis* | holotype, male | Durban, South Africa | photo | OUM |
| *schoetedeni* | *A. schoetedeni* | holotype, sex unknown | Boma, DR Congo | photo, pinned specimen | MRAC |
| *tenella* | *A. tenella* | lectotype, male | South Africa | photo, pinned specimen | ZMB |
| *tenella* | *A. tenella* | paralectotype, female | South Africa | photo, pinned specimen | ZMB |
| *tenera* | *A. tenella* (syn.) | type, sex unknown | Nairobi, Kenya | photo | MNHN |
| *umbripennis* | *A. navasi* (rep. name.) | holotype, female | Elizabethville, DR Congo | photo, pinned specimen | MRAC |
| *variolosa* | *A. tenella* **(syn. nov.)** | holotype, sex unknown | "Oceania" | photo | AMG, BMHN |
| *verruculata* | A. *verruculata* | type, sex unknown | Landjoro Pori, Kenya | photo | MNHN |
| *zonaria* | *A. zonaria* | holotype, male | DR Congo | photo, pinned specimen | MRAC |
| *zonata* | *A. zonata* | holotype, sex unknown | Gaboon | photo, pinned specimen | MRAC |
| *Mantispa* |  |  |  |  |  |
| *aphavexelte* | *M. aphavexelte* | holotype, male | Greece | literature, (Aspöck and Aspöck 1994) | HUAC |
| *aphavexelte* | *M. aphavexelte* | various specimens | Western Europe | pinned specimens | ZMB |
| *pagana* | *M. styriaca* (syn.) | holotype, sex unknown | France | photo, (H. Aspöck et al. 1980). | MNHN |
| *styriaca* | *M. styriaca* | Various specimens, not types | Western Europe | Photos, pinned specimens, literature, (H. Aspöck et al. 1980) | ZMB, MNHN |

**References for appendices**

Aspöck H, Aspöck U, Hölzel H (1980) Die Neuropteren Europas. Eine zusammenfassende Darstellung der Systematik, Ökologie und Chorologie der Neuropteroidea (Megaloptera, Raphidioptera, Planipennia) Europas. 2 vol. Goecke und Evers, Krefeld, 495 + 355 pp.

Aspöck U, Aspöck H (1994) Zur Nomenklatur der Mantispiden Europas (Insecta: Neuroptera: Mantispidae). Annalen des Naturhistorischen Museums in Wein 96: 99–114.

Eltringham H (1932) On an extrusible glandular structure in the abdomen of Mnatispa styriaca, Poda (Neuroptera). Transactions of the Entomological Society of London 80: 103–105.

Enderlein G (1910) Klassifikation der Mantispiden nach dem Material des Stettiner Zoologischen Museums. Stettiner Entomologische Zeitung 71: 341–379.

Erichson WF (1839) Beiträge zu einer Monographie von Mantispa, mit einleitenden Betrachtungen über die Ordnungen der Orthopteren und Neuropteren. Zeitschrift für die Entomologie 1: 147–173.

Esben-Petersen P (1917) Neue und wenig bekannte Mantispiden. Arkiv för Zoologi 11: 1– 15.

Fabricius JC (1775) Systema entomologiae, sistens insectorvm classes, ordines, genera, species, adiectis synonymis, locis, descriptionibvs, observationibvs. Flensbvrgi et Lipsiae, 832 pp.

Handschin E (1959) Beiträge zu einer Revision der Mantispiden (Neuroptera). I Teil. Mantispiden des Musee Royal du Congo Belge, Tervuren. Revue de Zoologie et de Botanique Africaines 59: 185–227.

Handschin E (1960) Beiträge zu einer Revision der Mantispiden (Neuroptera). II Teil. Mantispiden des “Musée Royal du Congo Belge”, Tervuren. Revue de Zoologie et de Botanique Africaines 62: 181–245.

Hoffman KM (2002) Family Mantispidae. In: Penny ND (Ed), A Guide to the Lacwings (Neuroptera) of Costa Rica. Proceedings of the California academy of Sciences, California, 251–275: 419–432.

Illiger JKW (1798) Verzeichnis der käfer Preussens, entworfen von Johann Gottlieb Kugelann à ausgearbeitet von Johann Karl Wilhelm Illiger. Mit einer vorrede des professors und pagenhofmeisters Helwig in Braunschweig, und dem ang. Halle: 510.

Lambkin KJ (1986) A Revision of the Australian Mantispidae (Insecta: Neuroptera) with a contribution to the classification of the Family I. General and Drepanicinae. Australian Journal of Zoology 1: 1–142.

Lambkin KJ (1986) A revision of the Australian Mantispidae (Insecta: Neuroptera) with a contribution to the classification of the family II. Calomantispidae and Mantispidae. Australian Journal of Zoology: 1–113.

Leach WE (1815) Entomology. In: Brewster D (Ed), Edinburgh Encyclopaedia. Edinburgh, 57–172.

Machado RJP, Rafael JA (2010) Taxonomy of the Brazilian species previously placed in Mantispa Illiger, 1798 (Neuroptera: Mantispidae), with the description of three new species. Zootaxa 2454: 1–61.

Makarkin VN (1985) K faune setchatokrylykh (Neuroptera) Dalnego Vostoka. Akademiia nauk SSSR. Zoologicheskii Zhurnal 64: 620–622.

Makarkin VN (1990) Novye setchatokrylye (Neuroptera) iz verkhnego mela Azii [Text in Russian.]. In: Akimov IA (Ed), Novosti faunistiki i sistematiki. Sbornik nauchnyhk trudov. Naukova Dumka Publ, Kiev, 63–68.

Monserrat VJ (1985) Lista de los tipos de Mecoptera y Neuroptera (Insecta) de la collección L. Navás, depositados en el Museo de Zoología de Barcelona. Miscellània Zooloògica 9: 233– 243.

Monserrat VJ (2014) Revisión de los mantíspidos de la Península Ibérica y Baleares (Insecta, Neuropterida, Neuroptera, Mantispidae). Graellsia 70: e012.

Navás L (1914)a) Algunos Neuropteros del Museo de Oxford. I serie. Boletìn de la Sociedad Aragonesa de Ciencias Naturales 13: 61–68.

Navás L (1914)b) Mantíspidos nuevos (Segunda [II] serie). Memorias de la Real Academia de Ciencias y Artes de Barcelona 3: 83–103.

Navás L (1914)c) Neuroptera nova africana. I series. Memorie dell’Accademia Pontifica dei Nuovi Lincei, Rome 32: 81–90.

Navás L (1914)d) Neurópteros de Oceania. Segunda [II] serie. Revista de la Real Academia de Ciencias Exactas Fisicas y Naturales de Madrid 12: 645–653.

Navás L (1914)e) Neuropteros nuevos de Africa. Memorias de la Real Academia de Ciencias y Artes de Barcelona 10: 627–653.

Navás L (1914)f) Notes sur quelques Nevropteres du Congo Belge [I]. Revue de Zoologie Africaines 3: 365–377.

Navás L (1914)g) Voyage de Ch. Alluaud et R. Jeannel en Afrique Orientale (1911-1912). Résultats scientifiques. In: Planipennia et Mecoptera. Paris, 52.

Navás L (1922) Insectos nuevos o poco conocidos [I]. Memorias de la Real Academia de Ciencias y Artes de Barcelona 17: 383–400.

Navás L (1923) Insecta nova. X Series. Memorie dell’Accademia Pontifica dei Nuovi Lincei, Rome 6: 19–27.

Navás L (1925) Névroptères nouveaux. Annales de la Société Scientifique de Bruxelles 44: 566–573.

Navás L (1927) Veinticinco formas nuevas de insectos. Boletín de la Sociedad Ibérica de Ciencias Naturales 26: 48–75.

Navás L (1929) Insectes du Congo Belge (Série III). Revue de Zoologie et de Botanique Africaines 18: 92–112.

Navás L (1930) Spedizione di S. A. R. Il Duca degli Abruzzi alle sorgenti dell’Uebi Scebeli – Risultati Zoologici. Descrizione di una nuova Mantispilla. Annali del Museo Civico di Storia Naturale Giacomo Doria, Genoa 55: 27–28.

Navás L (1933) Neurópteros exóticos [1.a serie]. Memorias de la Real Academia de Ciencias y Artes de Barcelona 23: 203–216.

Navás L (1936) Insectes du Congo Belge. Série IX. Revue de Zoologie et de Botanique Africaines 28: 333–368.

Neave SA (1939) Nomenclator Zoologicus. 7 vols. Edwards MA, Vevers HG (Eds). Zoological Society of London, London.

Ohl M (2004) Annotated catalog of the Mantispidae of the world (Neuroptera). International contributions on Entomology 5: 1–134.

Oliver GA (1797) Mante [= Mantis]. In: Encyclopedie Méthodique. Histoire Naturelle, Insectes. Vol. 7. Paris, 616–642.

Pallas PS (1772) Genus Mantis. In: Spicilegia zoologica, quibus novae imprimis et obscurae animalium species iconibus, descriptionibus atque commentariis illustrantur, cura P. S. Pallas. Tome 1, Fasciculus 9. Berolini, 12–15.

Penny ND (1982) Review of the generic level classification of the New World Mantispidae (Neuroptera). Acta Amazonica 12: 209–223.

Poda N (1761) Insecta musei Graecensis, quae in ordines, genera et species juxta systema naturae Caroli Linnaei digessit. Graecii: 127.

Poivre C (1981)a) Mantispidae du Cameroun II. Nouvelle description et Morphologie Externe comparee de Sagittalata lugubris et S. jucunda (Neuroptera, Planipennia). Neuroptera International 1: 110–121.

Poivre C (1981)b) Mantispides nouveaux d’Afrique et d'Europe (Neuroptera, Planipennia) (1re partie). Neuroptera International 1: 175–205.

Poivre C (1982) Mantispides nouveaux d’Afrique et d'Europe (Neuroptera, Planipennia) (seconde partie). Neuroptera International 2: 3–25.

Poivre C (1983) Morphologie externe comparee des Perlamantispa du sud de l’Europe: Perlamantispa perla (Pallas, 1772) et P. icterica (Pictet, 1865) (Planipennia, Mantispidae). Neuroptera International 2: 129–143.

Rafinesque CS (1815) Analyse de la nature ou tableau de l’univers et des corps organisés. Palerme: 224.

Scopoli JA (1763) Entomologia Carniolica, exhibens insecta Carnioliae indigena et distributa in ordines, genera, species, varietates, methodo Linneana. Trattner, Vindobonae, 420 pp.

Snyman L, Ohl M, Mansell M, Scholtz C (2012) A revision and key to the genera of Afrotropical Mantispidae (Neuropterida, Neuroptera), with the description of a new genus. ZooKeys 93: 67–93. doi: 10.3897/zookeys.184.2489

Snyman LP, Ohl M, Mansell MW, Scholtz CH (2012) A revision and key to the genera of Afrotropical Mantispidae (Neuropterida , Neuroptera), with the description of a new genus. ZooKeys 184: 67–93. doi: 10.3897/zookeys.184.2489

Walker F (1860) Characters of undescribed Neuroptera in the collection of W.W. Saunders. Transactions of the Entomological Society of London 5: 176–199.

Westwood J (1852) On the Genus Mantispa, with descriptions of various new species. Transactions of the Entomological Society of London 1: 252–270.
